# E-learning preferences of European junior neurologists—an EAYNT survey

**DOI:** 10.3389/fneur.2012.00167

**Published:** 2012-12-04

**Authors:** Laszlo K. Sztriha, Edina T. Varga, Krisztina Róna-Vörös, Natalja Holler, Raluca Ilea, Xenia Kobeleva, Cristian Falup-Pecurariu, Walter Struhal, Johann Sellner

**Affiliations:** ^1^Department of Clinical Neuroscience, King's College LondonDenmark Hill, London, UK; ^2^Institute of Genomic Medicine and Rare Disorders, Semmelweis UniversityBudapest, Hungary; ^3^Department of Neurology, Kecskemet County HospitalKecskemét, Hungary; ^4^Department of Neurology, University Medical Centre RegensburgRegensburg, Germany; ^5^Department of Neurology, Faculty of Medicine, Transilvania UniversityBrasov, Romania; ^6^Department of Neurology, Medizinische Hochschule HannoverHannover, Germany; ^7^Department of Neurology, General Hospital of the city of LinzLinz, Austria; ^8^Department of Neurology, Klinikum rechts der Isar, Technische Universität MünchenMünchen, Germany; ^9^Department of Neurology, Christian-Doppler-Klinik, Paracelsus Medizinische UniversitätSalzburg, Austria

## Introduction

Neurology is a fast advancing discipline in which professional development and improved patient care are much influenced by information technology. An important component of this is the ever increasing use of the internet for clinical, educational, and research activities, which also contributes to the requirement for health practitioners to keep their knowledge up-to-date (Masters, 2008; Gruener, 2010). The younger generation of physicians, particularly those born in or after the mid-1980s who have grown up in the internet era, reportedly use web resources more frequently and also as educational tools (Struhal et al., 2011). These individuals are more likely to be influenced in their clinical decisions by information found on the web (Romano et al., 2012).

There is a variety of electronic information resources for health practitioners to update their professional knowledge. A greater insight into the current use of web-based learning would not only shed light on the approach individuals take, but also help in determining necessary measures to improve the provision of electronic content. The objective of this study was to explore some aspects of the e-learning activity of European junior neurologists.

## Materials and methods

An anonymous multiple choice question based survey was conducted using a real time interactive voting system among the participants of a focused workshop organized by the European Association of Young Neurologists and Trainees (EAYNT) at the 2011 Meeting of the European Neurological Society (ENS). The questions addressed the frequency of internet use to specifically learn neurology, the most commonly assessed resources, the verification of credibility, and the opinions about websites aimed for patients. Seventy attendees participated.

## Results

**Figure 1:**
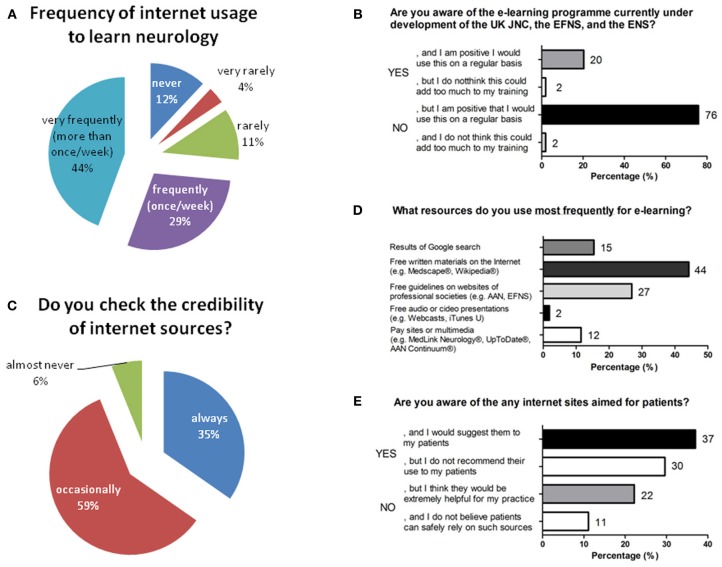
**Current e-learning preferences of 70 European junior neurologists. (A)** Frequency of internet usage to learn neurology. **(B)** Awareness for e-brain, the e-learning platform under development of the United Kingdom Joint Neurosciences Council (UK JNC), the EFNS and the ENS. **(C)** Usage of different resources for e-learning. **(D)** Assessment of credibility of internet sources. **(E)** Awareness of internet websites aimed for patients.

Demographic data revealed that 84% of the 70 participants were aged between 25 and 35 years. Most participants reported that, internet is used specifically to learn neurology at either at an average frequency of once weekly or more than once a week (Figure 1A). The most frequently accessed web resources are free written educational materials (e.g., Medscape®, Wikipedia®). Further sources include free guidelines published on websites of professional societies [e.g., American Academy of Neurology (AAN), European Federation of Neurological Societies (EFNS)], results of Google search, and pay sites or multimedia applications (e.g., MedLink Neurology®, UpToDate®, AAN Continuum®), as shown in Figure 1B. Free audio or video presentations (e.g., webcasts, iTunes U) were less preferred. More than one-third of the participants always verify the credibility of internet sources and two-third do this only occasionally or never (Figure 1C). Level of awareness about the e-learning platform eBrain, which was still under development at the time of the survey, was generally low (Figure 1D). Most junior neurologists are familiar with patient-oriented websites, but only a minority would recommend them to their own patients (Figure 1E).

## Discussion

E-learning includes the use of Internet-based approaches to broaden knowledge and improve performance. There is evidence for the effectiveness and acceptance of e-learning within the medical community (Ruiz et al., 2006). In this survey we confirm the widespread use of Internet in professional neurology education. While, there is a broad range of potential solutions on the Internet, the main resources remain free written materials including peer reviewed (e.g., Medscape®) but also non-peer reviewed ones such as those written by users (e.g., Wikipedia®). The latter is frequently used by clinicians likely owing to high Google hits, despite known shortcomings in breadth and occasional errors (Von Muhlen and Ohno-Machado, 2011). It is therefore, crucial to assess the credibility of the individual source, although only one-third of the participants indicated such an effort on a regular basis. Guidelines of professional societies are important learning materials, whereas pay sites have not gained significant importance in e-learning among junior neurologists. This may change with increasing access to eBrain, the world's largest, most comprehensive web-based training resource in clinical neuroscience (Holmes, 2012). However, while this program had been promoted for almost 2 years *prior* to the launch in late 2011, awareness was still relatively low just a few months before going live. Interestingly, although two-third were aware of patient websites, many would not recommend them to their patients.

While this study has several limitations, the findings underscore the importance e-learning has gained over recent years. Training to improve skills in assessing the credibility of web-based resources as well as better access to valuable high-end pay sites need to be considered as next steps in this development. It would also be relevant to further analyze the cause of negative perceptions about patient-oriented web content.

## References

[B1] Gruener G. (2010). Challenges for education. Front. Neurol. 1:12. 10.3389/fneur.2010.00012 21188252 PMC3008917

[B2] Holmes D. (2012). ebrain brings the e-learning revolution to the neurosciences. Lancet Neurol. 11, 126–127 10.1016/S1474-4422(12)70009-1 22265208

[B3] Masters K. (2008). For what purpose and reasons do doctors use the Internet: a systematic review. Int. J. Med. Inform. 77, 4–16 10.1016/j.ijmedinf.2006.10.002 17137833

[B4] Romano M. Gesualdo F. Pandolfi E. Tozzi A. E. Ugazio A. G. (2012). Use of the internet by Italian pediatricians: habits, impact on clinical practice and expectations. BMC Med. Inform. Decis. Mak. 12:23 10.1186/1472-6947-12-23 22455671 PMC3350423

[B5] Ruiz J. G. Mintzer M. J. Leipzig R. M. (2006). The impact of E-learning in medical education. Acad. Med. 81, 207–212 16501260 10.1097/00001888-200603000-00002

[B6] Struhal W. Falup-Pecurariu C. Sztriha L. K. Grisold W. Sellner J. (2011). European Association of Young Neurologists and Trainees: position paper on teaching courses for Generation Y. Eur. Neurol. 65, 352–354 10.1159/000327696 21625140

[B7] Von Muhlen M. Ohno-Machado L. (2011). Reviewing social media use by clinicians. J. Am. Med. Inform. Assoc. 19, 777–781 10.1136/amiajnl-2012-000990 22759618 PMC3422846

